# The effects of Gilles de la Tourette syndrome and other chronic tic disorders on quality of life across the lifespan: a systematic review

**DOI:** 10.1007/s00787-016-0823-8

**Published:** 2016-02-15

**Authors:** Joel Evans, Stefano Seri, Andrea E. Cavanna

**Affiliations:** 1 Department of Neuropsychiatry, BSMHFT and University of Birmingham Medical School, Birmingham, UK; 2 School of Life and Health Sciences, Aston University, Birmingham, UK; 3 Sobell Department of Motor Neuroscience and Movement Disorders, UCL and Institute of Neurology, London, UK

**Keywords:** Gilles de la Tourette syndrome, Tics, Behavior, Quality of life, Children, Adults

## Abstract

Gilles de la Tourette syndrome (GTS) and other chronic tic disorders are neurodevelopmental conditions characterized by the presence of tics and associated behavioral problems. Whilst converging evidence indicates that these conditions can affect patients’ quality of life (QoL), the extent of this impairment across the lifespan is not well understood. We conducted a systematic literature review of published QoL studies in GTS and other chronic tic disorders to comprehensively assess the effects of these conditions on QoL in different age groups. We found that QoL can be perceived differently by child and adult patients, especially with regard to the reciprocal contributions of tics and behavioral problems to the different domains of QoL. Specifically, QoL profiles in children often reflect the impact of co-morbid attention-deficit and hyperactivity symptoms, which tend to improve with age, whereas adults’ perception of QoL seems to be more strongly affected by the presence of depression and anxiety. Management strategies should take into account differences in age-related QoL needs between children and adults with GTS or other chronic tic disorders.

## Introduction

Chronic tic disorders encompass a continuum of childhood-onset neurodevelopmental conditions, ranging from the more severe Gilles de la Tourette syndrome (GTS) to chronic motor or vocal tic disorder. GTS is characterized by multiple motor and vocal/phonic tics which affects 0.3–1 % of the general population and is 3–4 times more common in males [1]. Tics are defined as sudden, rapid, non-rhythmic, involuntary movements (motor tics) or vocalizations (phonic tics); although there is typically a peak in severity in early adolescence, tics tend to vary in frequency and severity throughout life [2]. Despite empirically validated treatment strategies, a considerable proportion of patients irrespective of age fail to respond to either behavior therapy or pharmacological treatment and continue to experience significant symptom burden throughout life [3]. GTS is recognized as a complex disorder, being associated with co-morbid conditions such as obsessive–compulsive disorder (OCD), attention-deficit and hyperactivity disorder (ADHD), anxiety and affective disorders in around 90 % of patients according to both clinical and community studies [4–7].

Patients with GTS and other chronic tic disorders perceive their quality of life (QoL) as poorer than that of healthy individuals [8–11]. Understandably, both the direct consequences of tic expression and the efforts related to their suppression can present a functional burden for those affected. Moreover, research conducted over the last 15 years has highlighted that the presence of co-morbid behavioral problems can also be associated with poorer QoL, particularly in children [12]. However, evidence regarding the role played by tic severity or the specific QoL domains which are mostly affected has been inconsistent [13–17], partly due to the considerable variability in the instruments used to assess QoL throughout the years. Generic QoL measures are unlikely to be sensitive to specific features which are central to the perceived well-being of patients with GTS and other chronic tic disorders, and the recent introduction of a disease-specific instrument (GTS-QOL) facilitated the development of a fruitful line of research in this field [18–20].

While current evidence suggests that severity and frequency of tics may decline after childhood, the knowledge gap on determinants of QoL in GTS and other chronic tic disorders has been only partially filled by focused research in recent years [12]. Moreover, the differing natural course of tics and co-morbid behavioral symptoms can complicate the evolving picture of QoL in patients with these conditions [21–23]. An improved understanding of the specific domains of QoL which are affected throughout the lifespan will provide important information for strategic prioritization in clinical practice and resource allocation by healthcare providers in pediatric versus adult setting. Knowledge about QoL trends in GTS and other chronic tic disorders across the lifespan will also provide patients with useful information about the expected long-term outcomes of their condition. We, therefore, set out to explore possible differences in QoL domains across different age groups of patients with GTS and other chronic tic disorders by comprehensively reviewing the existing literature.

## Methods

The present systematic review was conducted in accordance with the methodology outlined in the Preferred Reporting Items for Systematic Reviews and Meta-Analyses (PRISMA) consensus statement [24]. Three electronic databases (PubMed, PsycInfo and PsycARTICLES), plus the NHS Evidence website, were searched using the following terms or MeSH headings: “Tourette”, “tic disorder”, “quality of life” and “functional impairment”. The same search terms were also used to identify relevant grey literature through Google Scholar, and the reference lists of articles which met selection criteria were manually screened for further relevant studies. All searches were restricted to publications in English language and availability of full text. Articles were not restricted by age, gender or other demographical criteria. Only studies in which patients received a formal diagnosis of GTS or other chronic tic disorders by an experienced clinician according to validated criteria were considered for inclusion. Childhood studies were defined as those with a maximum participant age of 18 years and a maximum mean age of 16 years. Studies which investigated patients with co-morbidities were considered eligible if QoL assessment was established as primary outcome measure. All studies using original quantitative research methodologies were eligible for inclusion in this review (retrospective/prospective cohort studies, cross-sectional studies and case series). Intervention studies were excluded as the outcome of QoL would have been evaluated based on the efficacy of an active intervention rather than the effects of GTS itself. Qualitative research was excluded as results would not be suitably compared to quantitative data within the present review [25].

The selected studies were assessed for methodological quality prior to inclusion using the Crowe Critical Appraisal Tool (CCAT). This instrument has shown appropriate construct validity in evaluating methodological quality and higher reliability than informal appraisal [26–28]. The properties of the CCAT allowed us to include a wide variety of study designs within the published literature [29]. Minimum standards for inclusion in our review were established following the author’s recommendations such that all appraised studies exceeded a minimum threshold quality score of 30 % [27].

## Results

**Fig. 1:**
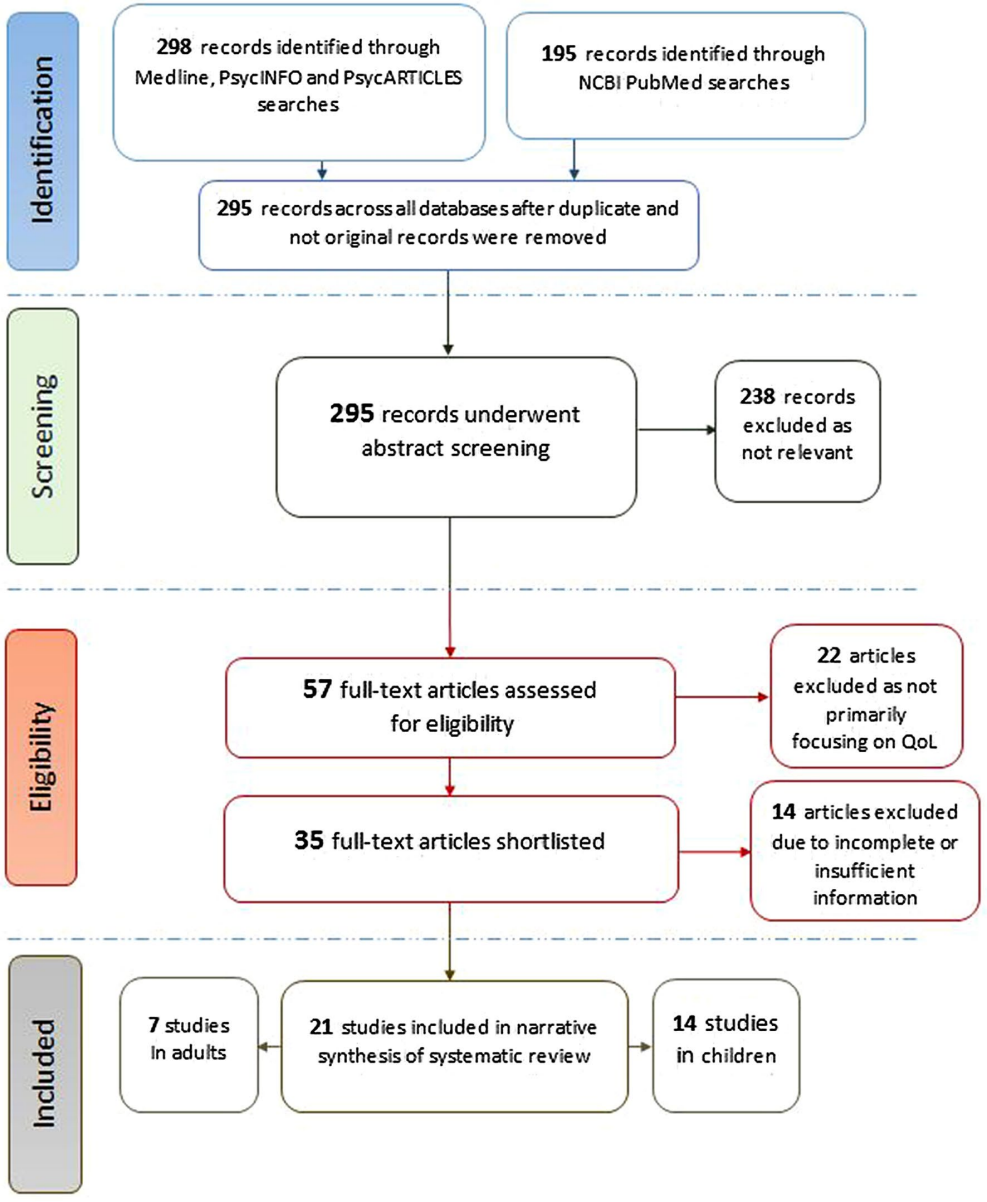
Flowchart outlining the study selection process

Our search strategy yielded a total of 57 relevant articles after duplicates were removed. A flow diagram which outlines the selection process is illustrated in Fig. 1.

**Fig. 2:**
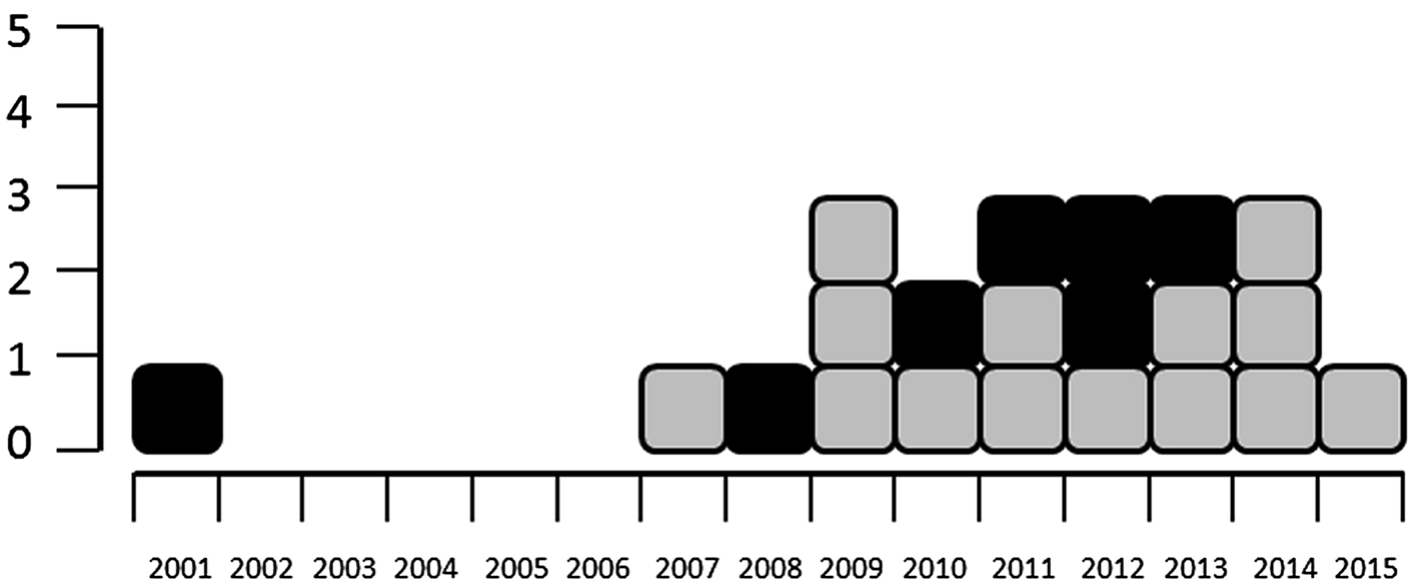
Studies investigating QoL in children (*grey squares*) and adults (*black squares*) with GTS or other CTDs, by year of publication

A total of 21 studies focussing on the QoL of patients with GTS or other chronic tic disorders met the inclusion criteria of this systematic review, 14 of which were conducted in children [14, 16, 17, 19, 30–39] and 7 in adults [15, 20, 40–44]. The vast majority of studies (20/21) were published during the last decade (Fig. 2).

A further 14 studies were deemed to be relevant; however, they had to be excluded due to incomplete or insufficient information [13, 18, 45–55]. The included studies were assigned a Quality Score through the standardized assessment of methodological quality, and were summarized in two separate tables according to child or adult target population (Tables 1, 2, respectively).

**Table 1:** Summary of published studies investigating QoL in children with GTS or other CTDs

Study/Country/setting	*n*	Mean age (range)/male gender (%)	Diagnosis (% co-morbidity)	QoL instrument (type)/subscales	QoL domains most affected	QoL domains least affected	Other findings	Quality score (%)
Storch et al. [30]/USA/University-based clinic	59	11.4 (8–17)/69	GTS or CTDs (48 % +ADHD; 42 % +OCD)	PedsQL (self- and proxy-rated)/Physical health; Psychosocial health; Emotional functioning; Social functioning; School functioning	Psychological; Social; School	Physical	Stronger parent–child agreement for QoL ratings in younger children (aged 8–11); Parent-rated school functioning likely overestimated due to co-morbid ADHD	63
Cutler et al. [31]/UK/University-based clinic	57	11.4 (8–17)/80	GTS (32 % +ADHD; 18 % +OCD)	PedsQL (self-rated)/Physical health; Psychosocial health; Emotional functioning; Social functioning; School functioning	Physical; Psychological; Social; School	–	Children’s reports of struggling to fit into society’s expectations; Psychological domain linked to school due to bullying	83
Pringsheim et al. [16]/USA and Canada/University-based clinic	71	11.2 (7–17)/79	GTS (56 % +ADHD; 41 % +OCD)	CHQ (proxy-rated)/Role limitation; Psychosocial score; Bodily pain; Physical summary; General health perception; Mental health; Self-esteem; Family activities	Social	Physical; Psychological	Impact on QoL linked to co-morbidities; Physical domain not affected in any clinical subgroup	63
Bernard et al. [17]/USA/University-based clinic	56	10.0 (5–17)/92	GTS (64 % +ADHD; 9 % +OCD)	TACQOL-PF (proxy-rated)/Body functioning; Motor functioning; Autonomy; Cognitive function; Social relationships; Emotional functioning	–	Physical; Psychological; Social; Cognitive	QoL affected by co-morbid ADHD and OCD but not tic severity	35
Hao et al. [32]/China/Paediatric Hospitals	424	−(8 to 12)/69	GTS (−)	PedsQL (self-rated)/Physical health; Psychosocial health; Emotional functioning; Social functioning; School functioning	Physical; Psychological; Social; School	–	QoL in GTS worse than in controls, but better than in patients with leukaemia; GTS characterized by better social functioning but worse school and emotional functioning than migraine/epilepsy	78
Eddy et al. [14]/Italy/University-based clinic	50	13.0 (10–17)/88	GTS (52 % +ADHD; 48 % +OCD)	YQOL-R (self-rated)/Relationships; Self; Environment; General	Physical; Psychological; Social	–	QoL differences in contextual items only; reduced social interaction and participation in activities; ‘Pure’ GTS more affected in environment domain, GTS + OCD in self/relationships domain	73
Conelea et al. [33]/USA/Internet-based survey	232	12.4 (10–17)/83	GTS or CTDs (37 % +ADHD; 39 % +OCD)	PedsQL (self-rated); FIQ (proxy-rated); TSIS (self-rated)/Physical health; Psychosocial health; Emotional functioning; Social functioning; School functioning; Negative family impact	Psychological; Social; School	Physical	Family functioning highlighted as relevant to QoL	80
Rizzo et al. [34]/Italy/University-based clinic	100	16.0 (13–18)/86	GTS (44 % +OCD; 3 % +ADHD + OCD)	YQOL-R (self-rated)/Relationships; Self; Environment; General	–	Physical; Psychological; Social	10-year follow-up revealed co-morbidity shift (increased OCD; decreased ADHD)	68
Cavanna et al. [19]/Italy/University-based clinic	75	12.0 (6–18)/80	GTS (8 % +ADHD; 33 % +OCD; 15 % +ADHD + OCD)	GTS-QOL-C&A (self-report)/Physical domain; Psychological domain; Obsessional domain; Cognitive domain	Physical; Psychological; Obsessional; Cognitive	–	Development and validation of first disease-specific QoL scale for adults with GTS (Italian language)	85
Cavanna et al. [35]/Italy/University-based clinic	75	12.0 (6–18)/80	GTS (8 % +ADHD; 33 % +OCD; 15 % +ADHD + OCD)	CTIM-P (proxy-report)/School activities; Home activities; Social activities	Social; School	–	Significant differences between parent and child reports; Parents may overestimate impact of co-morbidities versus tics on QoL	80
Liu et al. [36]/China/University-based clinic	107	10.1 (−)/86	GTS (8 % +ADHD; 32 % +OCD; 11 % +ADHD + OCD)	ISQL (self-rated)/Family life; Peer relationships; School life; Environment; Self-awareness; Experience of depression; Cognitive component	Psychological; Social; School; Cognitive	–	Influence of family stress and social withdrawal on QoL	63
Rizzo et al. [37]/Italy/University-based clinic	92	12.5 (7–17)/66	GTS (22 % +ADHD; 22 % +OCD; 6 % +ADHD + OCD)	YQOL-R (self-rated)/Relationships; Self; Environment; General	Psychological; Social	Physical	Influence of emotional lability on QoL	80
McGuire et al. [38]/USA/University-based clinic	24	11.3 (7–17)/75	GTS (42 % +ADHD; 38 % +OCD)	PedsQL (self-rated); CTIM-P (proxy-rated)/Physical health; Psychosocial health; Emotional functioning; Social functioning; School functioning; School activities; Home activities; Social activities	Physical; Psychological; Social; School	–	Differences in parent- and doctor-rated tic phenomenology	78
Gutierrez-Colina et al. [39]/USA/GTS summer camp	36	12.6 (8–18)/69	GTS (58 % +ADHD; 47 % +OCD)	PedsQL (self-rated)/Physical health; Psychosocial health; Emotional functioning; Social functioning; School functioning	Physical; Psychological; Social; School	–	No assessment of tic severity	68

*QoL* quality of life, *GTS* Gilles de la Tourette syndrome, *CTDs* chronic tic disorders, *ADHD* attention-deficit and hyperactivity disorder, *OCD* obsessive–compulsive disorder, *PedsQL* pediatric quality of life inventory, *CHQ* child health questionnaire, *TACQOL-PF* TNO-AZL children’s quality of life questionnaire-parent form, *YQOL-R* youth quality of life instrument-research version, *FIQ* family impact questionnaire, *TSIS* Tourette syndrome impact survey, *GTS-QOL-C&A* Gilles de la Tourette syndrome-quality of life scale (children & adolescents version), *CTIM-P* child Tourette’s syndrome impairment scale-parent report, *ISQL* inventory of subjective quality of life

**Table 2:** Summary of published studies investigating QoL in adults with GTS or other CTDs

Study/Country/Setting	*n*	Mean age (range)/Male gender (%)	Diagnosis (% co-morbidity)	QoL instrument (type)/subscales	QoL domains most affected	QoL domains least affected	Other findings	Quality score (%)
Elstner et al. [40]/UK/University-based clinic	103	29.0 (16–54)/68	GTS (21 % +OCD)	QOLAS (self-rated); SF-36 (self-rated)/Social functioning; Role limitation —emotional; Mental health; Cognitive functioning; Economic; Physical functioning; Role limitation —physical; Pain	Physical; Psychological; Social; Occupational; Cognitive	–	Reported difficulty in making/maintaining relationships; Effects of tics on employment status	63
Cavanna et al. [20]/UK/National TSA recruitment	136	25.9 (−)/72	GTS (51 % +ADHD; 32 % +OCD)	GTS-QOL (self-rated)/Physical domain; Psychological domain; Obsessional domain; Cognitive domain	Physical; Psychological; Obsessional; Cognitive	–	Development and validation of first disease-specific QoL scale for adults with GTS	73
Muller-Vahl et al. [15]/Germany/University-based clinic	200	35.0 (18–75)/75	GTS (–)	EQ-5D (self-rated)/Self-care; Usual activities; Pain and discomfort; Anxiety and depression; Mobility	Physical; Psychological	Physical (mobility only)	Higher anxiety/depression ratings compared to general population	75
Conelea et al. [41]/USA/Internet-based study	672	35.0 (18–77)/59	GTS or CTDs (23 % +ADHD; 35 % +OCD; 28 % +affective dis)	PQOL (self-rated); TSIS (self-rated)/Physical interference Social interference Cognitive health Occupational interference Psychological interference	Physical; Psychological; Social; Occupational; Cognitive	–	Impact of tics on occupation (7 % quit job; 13 % did not pursue job advancement)	85
Jalenques et al. [42]/France/Postal questionnaires	167	29.0 (16–68)/74	GTS (–)	WHOQOL-26 (self-rated); FSQ (self-rated)/Physical health; Psychological health; Social relationships; Environment; Physical functioning – ADL; Mental health; Work performance	Physical; Psychological; Social; Occupational	–	Impact of depression on QoL; quality of social interactions rated as very poor	78
Cavanna et al. [43] /UK/University-based clinic	46	24.0 (16–41)/89	GTS (54 % +ADHD; 30 % +OCD)	GTS-QOL (self-rated)/Physical domain; Psychological domain; Obsessional domain; Cognitive domain	Physical; Psychological; Obsessional; Cognitive	–	Childhood tic severity and family history of tics as predictors of poor QoL in adulthood	85
Crossley et al. [44]/UK/University-based clinic	72	26.0 (16–64)/65	GTS (22 % +ADHD; 18 % +OCD)	GTS-QOL (self-rated)/Physical domain; Psychological domain; Obsessional domain; Cognitive domain	Physical; Psychological; Obsessional; Cognitive	–	Impact of sensory phenomena on QoL	88

*QoL* quality of life, *GTS* Gilles de la Tourette syndrome, *OCD* obsessive–compulsive disorder, *ADHD* attention-deficit and hyperactivity disorder, *CTDs* chronic tic disorders, *QOLAS* quality of life assessment schedule, *SF-36* Medical outcomes study 36-item short-form health survey, *GTS-QOL* Gilles de la Tourette syndrome-quality of life scale, *PQOL* perceived quality of life scale, *TSIS* Tourette syndrome impact survey, *WHOQOL-26* World Health Organisation quality of Life 26-item scale, *FSQ* functional status questionnaire, *ADL* activities of daily living

The different aspects of QoL assessed in the reviewed literature could be categorized according to six recurring themes or domains, i.e. physical, psychological, occupational, social, cognitive, and obsessional aspects.

## Discussion

### Overall findings

GTS and other chronic tic disorders are lifelong disorders which broadly impact on QoL throughout their duration. Taken together, the results of the reviewed studies indicate that the perception of QoL may change significantly with age, in parallel with the natural course of the symptoms specific to the GTS spectrum. In children, the interaction between tics and co-morbid attention-deficit and hyperactivity symptoms can have a particularly severe impact on school life, whilst also having detrimental effects on the emotional, social and physical well-being which persist into adulthood. Adult patients with GTS or other chronic tic disorders tend to report a consistent global decline in QoL as a result of the persistence of tic symptoms, despite their reduced severity; moreover, the impact of co-morbid depression and anxiety on QoL seem to become more apparent with age. It is important to recognize the likely interplay between QoL domains, whereby for example specific components of emotional well-being such as decreased self-esteem and perceived stigma are likely to result in social withdrawal.

### Physical aspects

The physical domain of QoL reflected the presence of pain and injury as a direct result of tic severity. The results of the Tourette Syndrome Impact Survey, a study which involved both children and adults with GTS or other chronic tic disorders, showed that the majority of respondents reported at least one tic that caused pain or physical damage (64 and 60 %, respectively), with significant correlation to reported tic severity [33, 41]. Co-morbid ADHD and OCD symptoms were shown to further affect the physical aspects of QoL, especially in children [14, 17], with few exceptions [16]. In adults, difficulties in carrying out activities of daily living, including self-care, can cause significant distress as overt manifestations of problems in functional mobility and ability to perform exercises [56]. However, the overall preliminary findings from studies using disease-specific QoL measures suggest that perception of QoL is more strongly linked to physical health in children [19, 20]. Taken together, these results confirm that the physical components of QoL should not be overlooked throughout the lifespan.

### Emotional aspects

Emotional well-being is an important component of QoL classified under the psychological domain. Anxiety, feelings of frustration, hopelessness and low mood are commonly reported by patients with GTS or other chronic tic disorders and appear to be multifactorial in origin [42, 58]. A controlled study conducted in a clinical sample of children with GTS showed that anxiety and depression were significantly more prevalent than in both healthy children and patients with epilepsy [14]. Likewise, about 57 % of adult patients with GTS from a clinical sample reported problems with co-morbid anxiety and depressive symptoms, with an odds ratio of 13 compared to age-matched controls [15]. In general, psychological symptoms have been shown to be among the most important determinants of overall QoL [57], especially after the transition to adulthood [19, 20]. Interestingly, the results of the Tourette Syndrome Impact Survey indicated that children were considerably less likely than adults to believe that tics had led to the development of an emotional disorder (35 versus 59 %, respectively), despite awareness of stigma and isolation [33, 41].

### Occupational aspects

The negative impact of GTS and other chronic tic disorders for children in the school setting and for adults in the job environment was captured by the occupational domain of QoL. The presence of co-morbid behavioral problems, particularly ADHD, was consistently shown to affect school life [17, 30, 35] and overall QoL in children with GTS or other chronic tic disorders. The improvement of ADHD symptoms with age could contribute to explain the less pronounced impairment of QoL reported in adult working life [21, 22]. In fact, the results of the Tourette Syndrome Impact Survey showed that adults report milder interference with work productivity compared to the level of academic interference noted by children with GTS or other chronic tic disorders [33, 41]. Although the development of coping strategies throughout adolescence can subsequently result in improved satisfaction with life in the workplace [59], bullying and other distressing school experiences can have far-reaching consequences, possibly influencing future job choice and/or employment status [60, 61].

### Social aspects

Relationships with family and friends are key components of the social domain of QoL. Specifically, healthy family functioning has been recognized as integral to long-term social and emotional stability in children with GTS [62]. It has been shown that younger patients with tics can often feel responsible for family arguments as a result of their condition, and can be more likely to avoid communication with their parents [14, 16, 36], possibly resulting in increased insecurity and exacerbated problems over time [36]. Patients of all ages have reported higher interference from GTS and other chronic tic disorders within peer friendships than family relationships [33, 41], with potential difficulties in the formation of intimate or meaningful relationships which are an important part of adult life [42]; however, one study on adult patients showed that 29 % of participants felt unsupported by their family about their condition [40]. Although co-morbid depressive symptoms, emotional lability and anxiety were all identified as features of GTS and other chronic tic disorders potentially resulting in problems with social functioning [37, 57], the full extent of the impact of behavioral co-morbidities on social aspects of QoL remains difficult to determine, particularly in the case of adults with co-morbid OCD [12, 23].

### Cognitive aspects

Problems with concentration, forgetfulness and inability to complete important tasks encompass the cognitive domain of QoL. Interestingly, improvement of co-morbid ADHD symptoms with age seems to have a more significant impact on occupational than cognitive aspects of QoL [21]. A significant correlation between tic severity and cognitive domain scores was highlighted by the findings of the Tourette Syndrome Impact Survey [41]. Moreover, Studies conducted using the GTS-QOL further suggested that QoL perception can be more deeply affected by cognitive factors in adulthood than in childhood [19, 20]. These findings suggest that the interaction between tics and cognitive function in determining QoL across the lifespan is more complex than expected and deserves further investigation in future studies.

### Obsessional aspects

The development of disease-specific QoL measures for patients with GTS has enabled researchers to more sensitively assess the impact of repetitive behaviours and co-morbid OCD symptoms on the overall perception of QoL [19, 20]. Studies that have used the GTS-QOL report a decrease in the perceived impact of OCD on QoL from childhood to adulthood, despite the absence of clinically relevant decreases in OCD symptom severity, possibly reflecting the development of more effective coping strategies over time [34, 43]. Overall, disease-specific measures allow to more sensitively address the core symptoms of GTS and other chronic tic disorders compared to the generic measures that were used in 19 out of the 21 reviewed studies [63, 64].

### Methodological issues

In addition to the variability in study quality and research methodology, the reviewed literature contained a number of limitations that need to be taken into consideration when attempting to draw any conclusion. Participants in the reviewed studies were commonly recruited from tertiary referral centres, which usually recruit more severe and complex cases with a higher incidence of co-morbidities. This referral bias may limit the generalizability of the findings on the influence of GTS and other chronic tic disorders on QoL to the wider community of patients with these conditions. Moreover, there were inconsistencies in reporting self and proxy ratings of QoL in children with GTS and other chronic tic disorders, as well as co-morbidity rates across the lifespan. Specifically, in some studies parent-reported QoL was different from child reports [30, 65], raising the possibility that parent ratings might not capture the full extent of the effects of tics on the child’s QoL, especially with regard to subjective aspects. For example, none of the three reviewed studies that examined QoL of children with tic disorders by parent reports only demonstrated any deterioration in the psychological component of QoL [16, 17, 35]. Moreover, the role of treatment interventions for tics should be taken into account, as it can mitigate the impact of tic severity on QoL. For example, in the study by Bernard et al. [17], 39 % of the participants were receiving medication for their tics and tics were generally assessed to be under good control. Finally, our search methodology might have led to the exclusion of potentially relevant studies because of language or availability, resulting in reporting biases within the review process.

### Conclusions

The wide-ranging impact of GTS and other chronic tic disorders on the QoL of patients of all ages has been investigated in a number of dedicated studies since the new millennium. Research has mainly focused on the impact of tic symptoms and co-morbid behavioral problems on different QoL domains, which are characterized by varying degrees of functional overlap and potential interactions. Differences in QoL perception between children and adults suggest that a tailored approach could be the most fruitful strategy for the management of GTS and other chronic tic disorders across the lifespan. Future research using a longitudinal design is needed to further explore the natural history of tic disorders and associated behavioral co-morbidities, to determine their changing impact on QoL during the transition from childhood to adulthood. Finally, the use of disease-specific QoL measures in future studies will enable better understanding of QoL profiles in both clinic and community samples of patients with GTS and other chronic tic disorders.
